# F215. EARLY SIGNS ACTION PLAN: EXPERIENCES OF RELATIVES

**DOI:** 10.1093/schbul/sby017.746

**Published:** 2018-04-01

**Authors:** Nils Sjöström, David Berg, Sofia Johansson, Eva Andreasson, Marda Waern

**Affiliations:** 1 Sahlgrenska University Hospital; 2 Sahlgrenska University Hospital, University of Gothenburg

## Abstract

**Background:**

The shift towards person-centered care is ongoing within healthcare today. The Early Signs Action Plan was developed to facilitate participation of patients and their next-of-kin in outpatient psychiatric services specialized in the treatment of persons with schizophrenia and similar disorders. The aim was to investigate relatives’ experiences regarding the activation of the action plan for their next-of-kin.

**Methods:**

The study is a qualitative interview study using a semi-structured interview guide. The interviews are conducted with relatives (anticipated N=10) to outpatients whose Early Signs Action Plan has been activated. Interviews are digitally recorded and transcribed verbatim. The material is analyzed with qualitative content analysis

**Results:**

Preliminary analysis based on the first six interviews suggests that relatives experience increased involvement in services as well as improved relations with care staff. Relatives felt a greater sense of security as they were more knowledgeable, and activation of the plan resulted in a more immediate response from service providers. However, some respondents expressed communication problems with staff, pointing to a need for improved flow of information and increased understanding of the situation. Some expressed a feeling of uncertainty related to lack of feedback from staff, as well as lack of continuity and limited inclusion in the care process. Results from the entire study will be presented

**Discussion:**

Early Signs Action Plan may constitute a useful tool for the involvement of relatives in psychiatric services. However, relatives pointed out several areas for improvement

